# Focus on Diffusion MR Investigations of Musculoskeletal Tissue to Improve Osteoporosis Diagnosis: A Brief Practical Review

**DOI:** 10.1155/2015/948610

**Published:** 2015-03-10

**Authors:** Silvia Capuani, Guglielmo Manenti, Riccardo Iundusi, Umberto Tarantino

**Affiliations:** ^1^CNR-IPCF UOS Roma Sapienza, Physics Department, “Sapienza” University of Rome, Italy; ^2^Department of Orthopaedics and Traumatology, PTV Foundation, “Tor Vergata” University of Rome, Italy; ^3^Department of Diagnostic and Interventional Radiology, Molecular Imaging and Radiotherapy, PTV Foundation, “Tor Vergata” University of Rome, Italy

## Abstract

Nowadays, a huge number of papers have documented the ability of diffusion magnetic resonance imaging (D-MRI) to highlight normal and pathological conditions in a variety of cerebral, abdominal, and cardiovascular applications. To date, however, the role of D-MRI to investigate musculoskeletal tissue, specifically the cancellous bone, has not been extensively explored. In order to determine potentially useful applications of diffusion techniques in musculoskeletal investigation, D-MRI applications to detect osteoporosis disease were reviewed and further explained.

## 1. Introduction

Since the early publications by Le Bihan [1] and Basser [2–4] a great number of papers have documented the ability of diffusion magnetic resonance imaging (D-MRI) to define and discriminate normal and pathological conditions in a variety of cerebral [5, 6], abdominal [7–10] and cardiovascular applications [11, 12]. The enormous importance of D-MRI techniques in clinical diagnostics is due to the fact that biological water in tissues is an endogenous molecular probe and its diffusion reflects tissue configuration at a microscopic level. Therefore, diffusion magnetic resonance (MR) images can measure water proton displacements, within and between tissues, by probing molecules motion on the micrometer length scale which is orders of magnitude smaller than the macroscopic MR image resolution (usually 1–3 millimeters, in clinical applications).

To date, however, the potential role of D-MRI in the diagnosis of musculoskeletal disorders has not been extensively explored [13]. This is due to different causes of various nature that we have focused and described here, on the base of our experience as interdisciplinary group consisting of academics researchers in physics, radiologist clinicians, orthopedic surgeons and clinicians operating in the field of musculoskeletal diseases.

In the past, technical restriction limited the use of D-MRI techniques to the brain [5, 6, 12]. However, during the last 10 years, with the improvement in scanners technology and the availability of new MR sequences, investigation of the musculoskeletal system was made possible [14–23]. By using “water diffusion imaging” and “musculoskeletal” as key words for literature search, about twenty reviews are found. Some of these are focused on D-MRI for differentiation of acute benign and neoplastic vertebral compression fractures [14–16], painful spinal disorders [17], musculoskeletal tumor imaging [18, 19], and spine, muscles, and peripheral nerves disorders [20]. Some others are focused on D-MRI in body [21] and in pediatric radiology [22, 23]. However, none of these reviews is specifically focused on water diffusion MRI to detect osteoporosis. On the other hand, by using the following key words for literature search, “water diffusion imaging” and “osteoporosis,” only one relevant review, published in 2010, is obtained [14]. In this review, only very few studies about vertebral marrow diffusion in osteoporosis are reported, which show contradictory results.

Recently, in parallel with technological development, a new point of view about the study of the bone marrow water in cancellous bone has been introduced. This new point of view provides an explanation of the previous contradictory results extending the water diffusion investigation to other skeletal sites in addition to the vertebral one.

From the above considerations, the need for this review, which includes and explains the previous and the last recent results on the basis of simple and intuitive considerations regarding the biophysical mechanism of water diffusion in the tissues. Recommendations and suggestions about practical steps of optimization closely related to the microstructural characteristics of the different skeletal sites are also reported.

Osteoporosis is a systemic and metabolic disorder characterized by a progressive reduction of mineral bone mass and microarchitectural deterioration of bone tissue, which increases the risk of bone fractures [24, 25]. The clinical diagnosis of osteoporosis is currently based on the quantification of bone mineral density (BMD) performed by dual-energy X-ray absorptiometry (DXA) [26]. Although DXA examination is economical and noninvasive, it is known to have reduced sensitivity and a low predictive value on patients' risk of reporting bone fracture [27, 28]. This lack of sensitivity is likely to be due to the partial information that BMD provides on cancellous bone characteristics, assessing exclusively its mineral component [27, 29]. Indeed, bone strength depends on BMD and bone quality [25, 30, 31]. Bone quality refers to topological properties of trabecular microstructure, bone turnover, and composition of bone marrow. These and other factors, such as collagen framework and cell viability [32], may contribute to determining bone strength and its resistance to fracture [29]. In this regard, magnetic resonance MR techniques allow investigation of both trabecular networks and bone marrow [25, 33–37] providing some additional information on the physiological and functional changes associated with osteoporosis [29].

In general, D-MRI techniques are more challenging when applied to extracranial tissues due to technical issues mainly related to magnetic susceptibility variation Δ*χ* and motion sensitivity [13]. In particular, water diffusion in bone marrow and cancellous bone is affected by strong internal magnetic field gradients (*G*_*i*_) generated by Δ*χ* [38, 39] and mainly localized at the interface between bone and water components [40].

The setting, optimization, and operation of D-MRI techniques in cancellous bone and muscle investigations are markedly different from conventional MRI of the musculoskeletal system primarily based on image contrast due to differences in tissues relaxation times: *T*_1_, *T*_2_, and *T*_2_^*^ [41, 42]. Therefore, D-MRI applications demand a greater level of knowledge of the diffusion fundamentals, attention, and forethought. Currently, thanks to the development of high performance magnetic field gradients, new diffusion sensitized sequences, and parallel imaging techniques, the main technical problems are drastically reduced. Moreover, a new point of view on human cancellous bone [40, 43], described as a porous system in which water experiences a regime of restricted diffusion in the space between fat and mineral bone in each pore, forces to develop new MRI protocols based on molecular diffusion of water to obtain new microstructural information for improving osteoporosis diagnosis.

The basic principles of diffusion and its assessment of D-MRI in musculoskeletal tissue were summarized here, together with a brief description about microstructural characteristics of musculoskeletal tissues. Subsequently, some strategies and suggestions to optimize D-MRI protocols for their potential applications in the diagnosis of osteoporosis were described.

## 2. Microstructural Characteristics of Musculoskeletal Tissues

The principal musculoskeletal tissues that are related to osteoporosis and can be investigated with D-MRI are bones [33–38], bone marrow, muscles, and fat [44–48].

Bone tissue is a complex biomaterial composed of a solid mineral matrix, which is filled by bone marrow (a soft interstitial material). The solid matrix is mainly constituted by mineral components while bone marrow is mainly composed by water and fat at different relative percentages. The relative concentration of each bone marrow component is dependent on both its anatomical skeletal location and the age of considered individuals [49]. Human bone may be classified as cortical or trabecular. The former is mainly present in the shaft of long bones and is much denser (with a porosity ranging from 5% to 10%) than cancellous bone. Conversely, cancellous bone is much more porous (with a porosity ranging from 50% to 90%), and it is metabolically more active. From a physical point of view, cancellous bone is a porous system that can be described as a solid with holes and cavities, that is, presenting connected void spaces, randomly distributed within a solid matrix. Moreover, water is more prevalent in the boundary zone while fat occupies primarily the central zone of each pore, as recently demonstrated [40]. Water in the boundary zones of pores is also due to the presence of the endosteum, a thin membrane (with size about 3–7 *μ*m) of soft tissue (principally water) comprised of a linear chain of cells that lines the medullary cavity [50]. Moreover, due to a biological division of the bone marrow compartment, granulocytes and other nonfat entities accumulate at the boundary of the bone marrow compartment adjacent to the endosteum. Pore sizes in human cortical bone are in the range of several nanometers to few micrometers, while pore sizes in cancellous bone are in the range 1 *μ*m–1 mm, where intertrabecular plates space ranges approximately from 100 *μ*m to 1-2 mm. On the other hand, the space between fat and bone in the boundary of each pore ranges from less than 1 *μ*m (when endosteum is damaged or absent due to pathologies) to 10–20 *μ*m [50] or more, in the case of trabecular disruption or bone marrow irradiation. Bone marrow located inside the central cavities of long bones is not forced in pores. From a microstructural point of view, both bone marrow in trabecular bone and bone marrow free in the larger bone cavities are soft tissues characterized by several particles of spherical/elliptical shape and average size ranging from 6 microns of red blood cells to approximately 100 microns of the fat globules. Adipose tissue is characterized by approximately the same kind of bone marrow fat [51]. Skeletal muscles are attached to and bring about the movement of the various bones of the skeleton. The whole muscle is enclosed in a sheath of connective tissue that folds inwards into the substance of the muscle to surround a large number of smaller bundles, called fasciculi. These fasciculi consist of still smaller bundles of elongated, cylindrical cells named fibres that are characterized by diameter size of about 10–20 *μ*m.

With the development of osteoporosis, a change of the aforementioned microstructures size occurs. Bone microarchitecture is rearranged or disrupted (such as conversion of plates to rods and loss of connectivity) [41, 52, 53] and bone marrow which fills the trabecular holes is altered [44–46, 54]. Specifically, an increase of the marrow fat that decreases the interstitial space size in which water diffuse and a microstructural deterioration with a pore enlargement that increases the interstitial space size and their interconnection occur [43]. These changes do not result equally along all skeletal sites. As an example, in calcaneus, with the onset of osteoporosis, no bone marrow increase is detected but only a trabecular bone deterioration is observed [43, 55]. Moreover, recent investigations show a correlation between the cancellous bone deterioration and the loss of muscle mass with a change of fibres diameter size, referred to as sarcopenia [47, 48].

## 3. Magnetic Resonance Diffusion: Introduction and Concepts

Diffusion is the process by which matter is transported from one part to another as a result of random molecular motion, also called Brownian motion [56]. This motion results from the thermal energy carried by diffusing molecules and it is characterized by a diffusion coefficient *D* that provides information about the behavior of the molecular motion which in turn depends on the characteristics of the medium in which the diffusion occurs. Therefore, *D* measurement of water molecules in human tissues, as a consequence of the interactions between molecules and obstacles that hinder and or restrict their motion, gives information about size, orientation, and shape of cellular structures [1, 3].

Currently, D-MRI is the only means able to measure and monitor the diffusion coefficient in vivo, with a completely noninvasive modality and without requiring exogenous contrast agents. MRI measurements of diffusion indirectly measure the displacements of diffusing molecules in one dimension.

In Gaussian diffusion approximation, the mean square displacement (MSD) of diffusing molecules is linearly proportional to the diffusion coefficient *D* and the time *t* during which the diffusion process is observed [56]:

(1) $$\begin{array}{c} \text{MSD}=2NDt, \end{array}$$

where *N* is the “dimensionality” of the space over which diffusion distances are measured.

By using relation (1), it is possible to evaluate the length scale *l* or the characteristic length probed by diffusing molecules:

(2) $$\begin{array}{c} l={\text{MSD}}^{1/2}. \end{array}$$

By using relation (1) and (2) at human body temperature, during typical diffusion times of about *t* = 50 ms, random water molecules move in a homogeneous and isotropic medium over distances around *l* = 30 *μ*m in the tridimensional case (i.e., *N* = 3) and around *l* = 15 *μ*m along a chosen spatial direction (i.e., *N* = 1). However, this is the case of free diffusion of bulk water, for which there are no barriers to molecules motion and *D* ≈ 3∗10^−3^ mm^2^/s.

**Figure 1:**
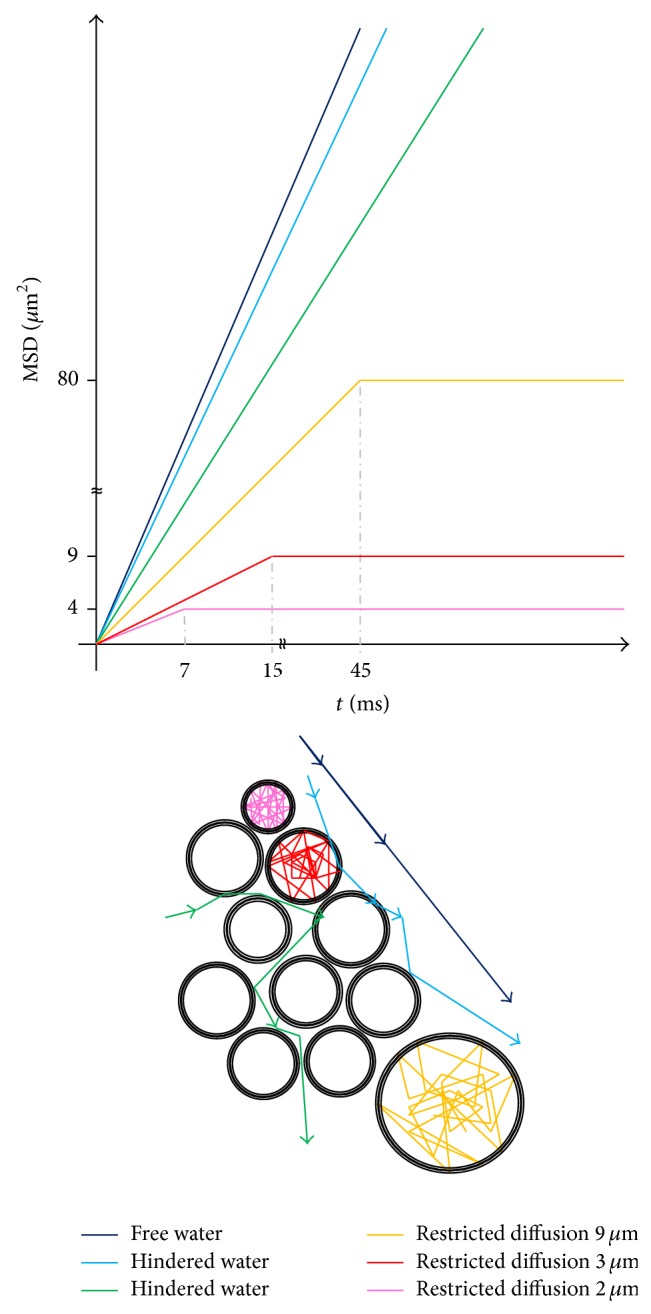
Schematic representation of water MSD as a function of the diffusion time *t*, obtained in a heterogeneous system depicted at the bottom. Solid lines represent curves obtained by using relation (1). In the case of restricted diffusion, the two solid lines represent curves obtained considering data at short and long times.

Because most human cells and subcellular structures have smaller dimensions than those listed above, it is highly likely that water molecules will have many interactions with cellular components over such *t* measurement interval [3]. As a consequence, water movement in tissues is neither entirely free nor random, being modified by interactions with hydrophobic lipid-containing cell membranes, intracellular organelles, and macromolecules and by flows within tubular channels such as blood vessels, capillaries, interconnected pores, and ducts. Water motion in tissue is therefore related to its microscopic and submicroscopic biological structure. As depicted in Figure 1, by using relation (1) and (2) and measuring *D*, it is possible to detect different water diffusion behavior, such as free, hindered, and restricted diffusion, in structures characterized by different size, and, more importantly, it is possible to estimate microstructures size. Specifically, if water diffusion is limited by a container (such as water in cortical bone pores, or intracellular water), when the molecules reach the boundaries, they are reflected and the MSD results confined in the container [56]. As a consequence, both *D* and *l* are lower than those of free bulk water.

## 4. Magnetic Resonance Diffusion Measurement: DWI and ADC

The molecular diffusion is uniquely assessed by diffusion weighted imaging (DWI) techniques [1, 3]. DWI techniques are able to measure the water diffusivity by the application of a couple of diffusion sensitizing gradients (motion probing gradients) to a simple *T*_2_-weighted spin-echo sequence, named pulse field gradient spin-echo (PFGSE) [57]. Signal losses on DWI are proportional to both diffusion coefficient of water molecules and the diffusion gradient strength used, as described by the signal attenuation *S*(*b*) of DWI, given by

(3) $$\begin{array}{c} S\left(b\right)=S\left(0\right)\exp\left(-bD\right), \end{array}$$

where *S*(0) is the signal amplitude in absence of diffusing gradients and b is the so-called *b*-value that indicates the strength and duration of application of diffusion sensitizing gradients together with the diffusion time *t* during which the diffusion phenomenon is observed:

(4) $$\begin{array}{c} b={\left(\gamma \delta g\right)}^{2}\left(\Delta -\frac{\delta }{3}\right), \end{array}$$

where *γ* is the gyromagnetic ratio, *g* is the strength of the gradient (diffusion gradient) used to encode diffusion, *δ* is the duration of the gradient pulse, and Δ, which is the time between the leading edges of the pair of diffusion gradient pulses in PFGSE, is the diffusion time *t* of relation (1). In the practice, the *b*-value which determines the degree of signal attenuation due to diffusion allows the selection of different diffusion behaviors characterized by different diffusion coefficients of water diffusing in heterogeneous systems (this issue will be discussed in paragraph 7).

By observing Figure 1, it is possible to understand that if the diffusion process is observed for time *t* which is too short, the diffusing water molecules do not reach obstacles and walls of pore; in this case, a free diffusion behavior and a free diffusion coefficient are detected. Therefore, an adequate diffusion time must be selected to obtain microstructural information from DWI investigations. As an example, to detect the presence of pores characterized by pore size of about 9 *μ*m, *t* greater than 45 ms must be used with *D* ≈ 3∗10^−4^ mm^2^/s, as shown in Figure 1. Moreover, free diffusion of water at human body temperature shows the highest *D* value approximately equal to 3∗10^−3^ mm^2^/s. Hindered and restricted water diffusion assume smaller values with increasing of obstacles and with the decreasing of pores and structures size within which the water is confined.

Because in anisotropic and heterogeneous systems diffusion coefficient depends on both the direction along which the diffusion gradient acts and the diffusion time *t* = Δ, DWI in tissues refers to ADC, the apparent diffusion coefficient, rather than *D*, the self-diffusion constant of a pure isotropic and homogeneous solution. By using relation (3), the ADC can be computed in each voxel of DW image to obtain an ADC map according to the following:

(5) $$\begin{array}{c} \text{ADC}=-\left(\frac{1}{b}\right)\ln\left[\frac{S\left(b\right)}{S\left(0\right)}\right]. \end{array}$$

## 5. Diffusion Tensor Imaging: DTI

Molecular mobility in tissues which are in general heterogeneous and anisotropic is not the same in all directions [2–4]. As an example, water diffusion along muscles fibers is less hindered than that along directions perpendicular to fibers [58]. In the presence of anisotropy, diffusion can no longer be completely characterized by a single scalar coefficient but required a tensor, $\underline{D}$, which fully describes molecular mobility along each direction and correlation between these directions. Because $\underline{D}$ is symmetric, only six ADC measurements along six independent directions are needed, and relation (3) is now expressed by the following [2]:

(6) SD_=S(0)exp⁡−bxxDxx−byyDyy−bzzDzzhhhhhhh−2bxyDxy−2bxzDxz−2byzDyz,

where, the on-diagonal terms *D*_*xx*_, *D*_*yy*_, and *D*_*zz*_ represent the ADC along axes *x*, *y*, and *z*, respectively, while the off-diagonal terms *D*_*xy*_, *D*_*xz*_, and *D*_*yz*_ reflect correlation between molecular displacements in perpendicular directions [2–4].

In practice, to determine $\underline{D}$, one must first collect DW images along at least six independent directions, by using diffusion sensitized MRI pulse sequences with diffusion gradients along six independent directions, such as *x*, *y*, *z*, *xy*, *xz*, and *zy*, plus a spin-echo image that provides the *S*(0) term in relation (6). Therefore, DTI protocol lasts six times more than the DWI protocol; however, from the tensor, several pieces of information can be extracted. The sun of the on-diagonal elements is rotationally invariant measure of diffusion, that is, a measure of diffusion independent on the reference frame. This is very important in diagnostic applications because diffusion quantification obtained at different times in a given patient or in different patients and healthy subjects or in different hospitals can be compared. One-third of the trace is often referred to as the mean diffusivity (MD) and constitutes a directionally averaged diffusion coefficient that can also be obtained by averaging the ADC measured along *x*, *y*, and *z* directions. The fractional anisotropy (FA), a sort of normalized standard deviation, measures the fraction of the magnitude of $\underline{D}$ that can be ascribed to anisotropic diffusion. FA varies between 0 (isotropic diffusion) and 1 (maximum anisotropy). As an example, biceps femoris short head in a healthy subject is characterized by FA = 0.4 [59], but the same tissue in the presence of edema shows FA values less than 0.3.

## 6. D-MRI Pulses Sequences

Diffusion gradients can be applied to many standard MRI sequences, but, particularly in musculoskeletal applications, the images produced are prone to artifacts resulting from physiological motions, eddy currents, and internal gradients due to Δ*χ*. Spin-echo DWI sequences such as pulse gradient spin-echo (PGSE) [57] or pulse gradient stimulated echo (PGSTE) [60] that is particularly useful to investigate diffusion in tissues characterized by *T*_2_ ≪ *T*_1_ (such as the most musculoskeletal tissues) yield high signal-to-noise ratio (SNR) images and are more resistant to field inhomogeneity created by the application of diffusion gradients. However, long acquisition times of several minutes limit their clinical use due to the increased likelihood of encountering motion-related artifacts.

Because the reliability of diffusion contrast depends on SNR of DWI images [61], it must be approximately higher than 2 [62] to exclude an underestimation of the MD and an overestimation of the anisotropy quantified by FA [62, 63].

Single shot echo planar imaging (SS-EPI) sequences offer significantly faster acquisition times, thus overcoming DWI sensitivity to patient movement while still offering a relatively high SNR.

A major limitation of diffusion sensitized SS-EPI imaging is magnetic susceptibility artifacts, at different tissue interfaces common in musculoskeletal imaging, such as bone and soft tissue, which can lead to severe geometric image distortion, relatively low spatial resolution due to rapid *T*_2_^*^ decay of signal, and in general low image quality.

Recent advances in SS-EPI to overcome these problems include improved gradient systems with reduced eddy current effects which can help reduce geometric distortions and parallel imaging techniques employing multiple receiver coil elements to improve acquisition times and increase spatial resolution.

An alternative approach is to acquire EPI data using a segmented or multishot, echo planar readout (MS-EPI), which divides the echo train into several shorter parts. This makes the image less sensitive to susceptibility artifacts, reduces image distortion, and increases spatial resolution but at the price of longer acquisition times and therefore at a greater risk of motion artifact.

Recently [64], by using a diffusion sensitized segmented EPI with EPI factor equal to 7 and a scan time duration for the DTI protocol equal to 17:50 minutes, we have obtained the first MD and FA maps of femoral neck performed in three different groups of women based on their BMD (healthy, osteopenic, and osteoporotic women) [64]. Each DW image, acquired by using four averaged scans, was characterized by a sufficient SNR to extract significant values of MD and FA and an in-plane resolution of about 2.2 mm with a slice thickness of 5 mm [64]. Subsequently, the same diffusion sensitized segmented EPI sequence has been used to evaluate ADC in calcanei of healthy and osteoporotic subjects [43, 55].

## 7. D-MRI Parameters for Optimization

The choice of pulse sequence parameters has a profound influence on the accuracy and precision in the estimates ADC, MD, and FA and other diffusion metrics parameters. Currently, the most of these sequence parameters are already preset in clinical scanner to perform brain, spinal cord, or muscles investigations. For example, it is well known that DTI protocol with *b* = 1000 s/mm^2^ is used in conventional neurological clinical applications [6, 7, 12], while *b* = 500–800 s/mm^2^ optimizes diffusion measurement in muscles [65–67] and spinal cord [68]. Conversely, no suggestions can be usually found to perform diffusion investigation in cancellous bone.

In principle, the optimal *b*-value is approximately equal to the reciprocal of ADC (or MD) that one is trying to estimate:

(7) $$\begin{array}{c} b\approx \frac{1}{\text{ADC}}. \end{array}$$

However, the best compromise between a sufficient SNR and a sufficient diffusion weighted contrast to detect diffusion of bone marrow water restricted between fat and bone must be reached.

**Figure 2:**
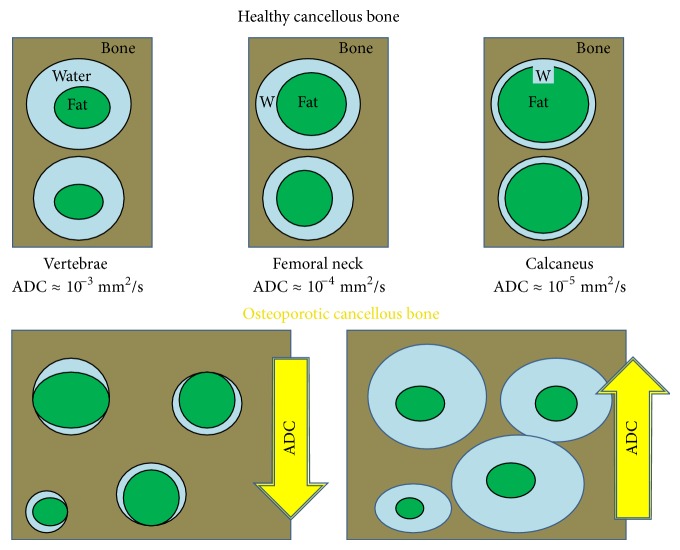
Schematic representation of cancellous bone at different skeletal sites in healthy (top) and osteoporotic (bottom) cancellous bone. Fat content increases from axial skeleton to peripheral skeletal sites. As a consequence, water ADC is higher in the vertebrae and lower in the calcaneus. Indeed, diffusion of water in calcaneus is more restricted compared to water in femoral neck or in vertebrae. With the development of osteoporosis, two different and opposite mechanisms occur: an increase of the marrow fat which decreases the ADC value and a microstructure deterioration with a pore enlargement that increases ADC. In the calcaneus, only the last mechanism results (adapted from reference [43] with permission).

Bone marrow fat extracted from the vertebrae, the femoral neck, and the calcaneus of healthy postmenopausal women was found to range from 50% to 70%, 60% to 88%, and 78% to 98%, respectively [40, 44, 45, 55, 64, 69–71]. An increase in the amount of bone marrow fat involves a decrease of the interstitial space between bone and fat (region in which water diffuses), leading to a lower water ADC. As a consequence, water ADC is higher in the vertebrae, intermediate in femoral neck, and lower in the calcaneus; that is, diffusion of water in the calcaneus is more restricted compared to water in femoral neck or in vertebrae, as displayed in Figure 2.

Therefore, these three different skeletal sites must be probed with different length scale (because they are characterized by different pores size) and different *b*-values, because they are characterized by different water ADC. Specifically, ADC values of bone marrow water measured in human cancellous bone of healthy subjects showed the following approximate values: ADC ≈ 10^−3^ mm^2^/s, ADC ≈ 4∗10^−4^ mm^2^/s, and ADC ≈ 5∗10^−5^ mm^2^/s for the vertebrae, the femoral neck, and the calcaneus, respectively [40, 43, 55, 64, 70, 72, 73]. In principal, by using relation (7), *b*-values around *b* = 1000 s/mm^2^, *b* = 2500 s/mm^2^, and *b* = 20000 s/mm^2^ should be used to measure ADC in vertebrae, femoral neck, and calcaneus, respectively. In reality, most tissues are made of multiple subcompartments (for example, the intra- and extracellular water compartments); each of these is characterized by a peculiar ADC. As a consequence, the ADC that is measured could depend on the range used for the *b*-values, as measurements with low *b*-values would be more sensitive to fast (near free or less hindered) diffusion components and measurements with high *b*-values would be more sensitive to slow (more restricted and entrapped) diffusion components. In the ideal case, a very large range of *b* values would be required. However, this means long acquisition times incompatible with clinical applications. In any case, it is clear that it is better to estimate the averaged ADC in a tissue by using a range of *b*-values (from four to eight) and a fitting procedure of experimental data to relation (3) rather than estimate it using a single *b*-value.

As previously underlined, in cancellous bone, more than in other tissues, the magnetic susceptibility mismatch between the solid matrix and interstitial water bone marrow generates internal gradients of magnitude depending on the geometry and orientation of the trabeculae with respect to the static magnetic field direction scaling with magnetic field strength. Therefore, the *G*_*i*_ strength that water molecules sense as they diffuse near the interface between fatty marrow and bone increases with the decreasing of the interstitial space and with the increasing of the static magnetic field [40, 55, 74]. In particular, when cancellous bone in calcaneus is investigated, a positive coupling between *G*_*i*_ and diffusion gradients occurs that produce an effective *b*-value higher than that computed by using relation (4). Finally, it is well known that the diffusive motion of water molecules through the *G*_*i*_ leads to irreversible dephasing [40, 55, 74–77]. As a consequence, the effective diffusion coefficient ACD_eff_ (measured by using a DWI protocol) results in smaller than the ideal ADC obtained without taking into account the  Δ*χ* at the interfaces between water and bone [55, 78, 79]. Because of the above considerations, particularly in calcaneus D-MRI investigations, water ADC is underestimated and the use of a *b*-value range up to approximately 10000 s/mm^2^ should be appropriate.

By considering relation (3) and (4), in order to achieve significant signal attenuation when the diffusion gradients are applied, the pulses must be strong. However, this places strong demands on the magnetic field gradient hardware. Therefore, D-MRI investigations in cancellous bone required high gradient unit performance of MRI scanner. The choice to select diffusion gradients applied for a long time (i.e., long *δ*) to increase diffusion gradients strength is not applicable in cancellous bone and muscles investigations. Indeed, water in bone marrow and muscles is characterized by lower *T*_2_, compared to other tissues (such as brain tissue). Therefore, long *δ* can lead to extended echo times, which consequently determines poor SNR in the resulting DW images. Moreover, due to the reduced *T*_2_ of water in musculoskeletal tissues, the use of PGSTE (which requires Δ < *T*_1_) rather than PGSE (which requires Δ < *T*_2_) sequences would be preferred.

The *b*-value includes both diffusion time Δ and diffusion gradients strength *g*. Therefore, one can obtain certain *b*-value ranges by fixing the diffusion time Δ and varying *g* or by fixing *g* and varying Δ. These two modalities do not provide equal investigations and equal results. This is because Δ also defines the length scale through which a tissue is probed while *g* only determines the degree of signal attenuation due to diffusion.

Recently [43, 55], ADC values of bone marrow water measured in calcaneus showed an increasing trend from healthy (ADC ≈ 4∗10^−11^ mm^2^/s) to osteoporotic subjects (ADC ≈ 7∗10^−11^ mm^2^/s) and a significant negative correlation between ADC and BMD values. This study, performed with *b* = 8000 s/mm^2^ and Δ = 30 ms, suggests that by using *l* ≈ 3 *μ*m healthy, osteopenic, and osteoporotic subjects can be significantly discriminated. This is most likely due to the fact that the spaces sizes between fat and bone (where the water diffuses) are around 3 *μ*m and they increase with the development of osteoporosis, according to the ADC increase [43, 55]. On the other hand, healthy and osteoporotic subjects were significantly discriminated by selecting *b* = 2500 s/mm^2^ and Δ = 40 ms, to probe femoral neck with *l* ≈ 10 *μ*m [64]. In this DTI study, both MD and FA values obtained in femoral neck showed a decreasing trend from healthy to osteoporotic subjects. These results suggest that, with the development of the osteoporosis, the space between fat and bone probed by water decreases due to marrow fat increases and it becomes more isotropic due to the structural rearrangement of the endosteum cell lines or of the trabeculae surface [64].

## 8. Conclusion

D-MRI in cancellous bone provides a new tool to probe tissue microstructures changes that occur with the development of osteoporosis. This is because at microscopic level many tissue features related to osteoporosis influence MR diffusion measurements.

Currently, diffusion in musculoskeletal tissue is quite difficult to use successfully, principally because of the occurrence of strong internal gradients due to magnetic susceptibility mismatch between soft tissue and mineral bone and short *T*_2_. These obstacles, however, can sometimes be overcome with ad hoc optimized pulse sequences, hardware, and a theoretical background of diffusion process.

## Abbreviations by Order of Appearance in the Paper

MR: Magnetic resonance
MRI: Magnetic resonance imaging
D-MRI: Diffusion magnetic resonance imaging
BMD: Bone mineral density
DXA: Dual-energy X-ray absorption
Δ*χ*: Magnetic susceptibility variation
*G*_*i*_: Internal magnetic field gradients
MSD: Mean square displacement
*D*: Diffusion coefficient
*l*: Length scale
DWI: Diffusion weighted imaging
DW: Diffusion weighted
PFGSE: Pulse field gradient spin-echo
*b*: *b*-value
*γ*: Gyromagnetic ratio
*g*: Strength of the magnetic field gradient
*δ*: Duration of the magnetic field gradient pulse
Δ: Diffusion time: the time between the pair of diffusion gradient pulses
ADC: Apparent diffusion coefficient
DTI: Diffusion tensor imaging
$\underline{D}$: Diffusion tensor
MD: Mean diffusivity
FA: Fractional anisotropy
PGSE: Pulse gradient spin-echo
PGSTE: Pulse gradient stimulated echo
*T*_2_: Transverse relaxation time
*T*_1_: Longitudinal relaxation time
SNR: Signal-to-noise ratio
EPI: Echo planar imaging
SS-EPI: Single shot echo planar imaging
MS-EPI: Multishot echo planar imaging.
